# Gender role attitudes and father practices as predictors of nonresident father-child contact

**DOI:** 10.1371/journal.pone.0266801

**Published:** 2022-04-21

**Authors:** Marieke Heers, Ivett Szalma

**Affiliations:** 1 Swiss Centre of Expertise in the Social Sciences (FORS), University of Lausanne, Lausanne, Switzerland; 2 Centre for Social Sciences, Hungarian Academy of Sciences Centre of Excellence, Cornivus University of Budapest, Budapest, Hungary; University of Michigan, UNITED STATES

## Abstract

Due to an increasing number of parental union dissolutions, a growing number of fathers does not cohabit with their biological children. This article analyses individual and societal gender role attitudes as well as societal father practices as determinants of nonresident father-child contact. Previous research shows that individual-level factors influence the relationship between nonresident fathers and their children. Research on resident fathers indicates that individual attitudes and societal contexts affect father-child involvement. Little is known on the relationship between individual gender role attitudes as well as societal gender role attitudes and father practices and nonresident fathers’ involvement in their children’s lives. To shed more light thereon, we examine data from eleven Eastern and Western European countries from the first wave of the Gender and Generations Survey. We analyze two samples: One consisting of nonresident fathers of children aged 0 to 13 and one of fathers of adolescents aged 14 to 17. Logistic regression models assess if individual and societal gender role attitudes as well as societal father practices predict the probability of monthly father-child contact. Contact between nonresident fathers is affected by different factors depending on whether the focus is on children or adolescents. Societal gender role attitudes and societal father practices predict the probability of monthly contact between fathers and their children; individual gender role attitudes are less important. Individual gender role attitudes, on the other hand, predict the probability of monthly contact between nonresident fathers and their adolescent children; societal factors matter less for this age group.

## 1. Introduction

Across countries, parental union dissolution has become increasingly common. While most children continue cohabiting with their mothers, most of them experience a decrease in contact with their fathers [1–8]. Prior research has assessed how fathers’ socio-demographic factors as well as children’s characteristics predict the frequency of nonresident father-child contact [9–11]. At the same time, fathers’ enactment of their father role depends on societal contexts and expectations towards them [7, 12, 13]. Yet, little is known about how societal contexts, attitudes and norms affect nonresident father-child relationships.

Societal norms towards fatherhood are changing. A ‘new fatherhood’ has emerged and fathers now more often want to perform interactive and caregiving activities and are also expected to do so [14–17]. Beyond socio-demographic characteristics, resident fathers’ involvement in childrearing is defined by their own gender role attitudes and by societal norms and expectations [18, 19]. The extent to which individual and societal gender role attitudes as well as societal father practices relate to nonresident father-child contact in the European context has not yet been established. Therefore, this study assesses the relationship between individual-level gender role attitudes and societal-level gender role attitudes and father practices with nonresident father-child contact.

Maintaining father-child contact after union dissolution is crucial as father involvement has positive benefits for children’s social, emotional, and intellectual development, their wellbeing, behavior and educational success [7, 20–27], as well as for fathers’ wellbeing [28] and psychological distress [29]. Furthermore, nonresident father-child contact is policy-relevant [4]. Father-oriented policies mainly relate to resident fathers spending more time with their children [30]. With respect to nonresident fathers it needs to be unfolded how to support them in remaining involved in their children’s lives [30–32].

Our study addresses two research questions: *How are individual- and societal gender role attitudes associated with nonresident father-child contact*? *How do societal father practices relate to nonresident father-child contact*? We use data from the Generations and Gender Program [GGP; 33, 34] from eleven European countries and rely on fathers’ reports. With the latter, we contribute considerably to the literature as despite the growing research and policy interest in fathers, data obtained directly from fathers is rare; this is even more the case for nonresident fathers [29, 35, 36].

Prior studies on nonresident father-child contact have mostly focused on single Anglo-Saxon and Western European countries. To broaden the evidence base, our study includes eight Eastern European and three Western European countries. Eastern and Western European countries differ considerably in terms of gender equality and family practices [37, 38]. For several decades, the level of gender equality has been significantly higher in Western than in Eastern European countries. The Gender Inequality Index clearly shows that gender inequality is more pronounced in Eastern than in Western European countries, while within both regions there is considerable heterogeneity in terms of gender equality [39]. Moreover, previous research points towards unequal of fathering practices in Eastern compared to Western European fathers, with fathers generally being less involved in Eastern than in Western European countries [30, 37, 40].

Previous studies on nonresident father-child contact have focused on children of specific age groups [28, 41], and have mostly focused on younger children. Here, we consider nonresident father-child contact for a sample of children (aged 0–13) and for a sample of adolescents (14–17) and, thereby, depict father involvement across childhood and adolescence. Prior research has established how gender role attitudes and societal expecations and norms relate to resident father child-relationhips. The aim of this study is to extend that literature on gender role attitudes and father practices of nonresident fathers.

## 2. Background

### 2.1. Gender role attitudes and father involvement

Gender role attitudes are a representation of what is considered appropriate for male and female roles in a given cultural context [42–44]. They range from traditional to egalitarian where traditional gender role attitudes refer to beliefs in gendered separate spheres of men and women in the employment and family domains [45, 46]. Fathers’ gender role ideology relates to their beliefs about appropriate fathering behaviors [47]. Multiple studies have assessed how resident fathers’ gender role attitudes determine the involvement with their children: The vast majority of these studies shows that fathers with more egalitarian gender role attitudes demonstrate more involvement with their children than fathers with more traditional attitudes, or–put differently–that fathers holding more traditional values are less involved with their children [45, 47–52]. Similarly, parents with more modern gender role attitudes are more likely to have a gender-equal division of childcare [51]. Research on resident fathers has also shown that once men are involved in looking after their children, that pattern continues over time [50]. When parents separate, this pattern is challenged.

While gender attitudes are an acknowledged factor in explaining resident fathers’ involvement in the home and the family [53], they have rarely been considered in the empirical literature on nonresident fathers. Cooksey and Craig (1998) have analyzed US-data and found that men who adhere to traditional gender role ideologies regarding the division of labor within families tend to have frequent contact with their nonresident children. This contradicts the empirical findings regarding resident fathers’ involvement referred to above, stipulating that men with nontraditional gender role-ideologies are more likely to participate in childcare. To shed more light on how individual gender role attitudes affect nonresident fathers’ involvement with their children, in the below analysis, we assess how nonresident fathers’ gender role attitudes relate to the involvement with their children when a large number of European countries is considered.

### 2.2. Societal gender role attitudes and father involvement

In addition to individual-level factors, cultural norms, contexts and preferences influence fathers’ involvement with their resident children [30, 54]. Given that gender role attitudes differ across countries [18, 39, 55, 56], one might expect that fathers living in societies characterized by traditional gender role ideologies are less involved in their children’s lives than fathers with more modern gender role ideologies. However, evidence on the relationship between societal gender role attitudes and nonresident father-child contact has only started to emerge. Kalmijn (57) uses the SHARE-data on ten countries and measures societal gender roles and finds that more traditional gender roles are negatively related to the intensity of contact between divorced fathers aged 50 and older and their adult children. The author attributes this association to the fact that when gender roles are more egalitarian, fathers invest more in their children; both during marriage and after divorce. Based on data from England, Germany, the Netherlands and Sweden, Kalmijn [28] finds that nonresident father child-contact after divorce is more common in countries with higher divorce rates and hypothesizes that gender role attitudes explain a large part of the cross-country variation in nonresident father-child contact. That study does not explicitly measure gender role attitudes. In the below analysis, we measure societal gender role attitudes and assess whether nonresident fathers living in societies characterized by traditional gender roles have less contact with their children than nonresident fathers living in societies with more egalitarian gender role attitudes.

### 2.3. New fatherhood, societal father practices and nonresident father-child involvement

During the last decades, fathers have taken up an increasingly prominent role in childrearing [58–60]. Accordingly, the concept of ‘new fatherhood’ has emerged [13, 14, 16, 61] and fathers are now more committed to be involved and to nurture their children and are also more emotionally involved than fathers were in the past [62]. On the one hand, this can be explained by growing normative pressures on fathers to be involved; on the other hand, fathers want to be active in childrearing [4, 63]. New fatherhood behaviours are also encouraged by policy incentives aimed at the involvement of fathers in childcare that are implemented across Europe [64]. Research also posits that men who rate fathering as more important and who have positive attitudes towards parenting are more involved with their children [12, 14, 59, 65–67].

Fathers’ involvement and new fatherhood behaviors are challenged when parents separate and most children continue living with their mothers [3, 6, 68]. However, a thorough understanding of the extent to which new fatherhood behaviors relate to separated families is lacking. A recent study on data from the UK shows that fathers who were more involved prior to parental separation are more involved after separation [8]. Carlson and McLanahan [69] found that parents’ ability to work together in rearing their common child helps keeping nonresident fathers connected to their children. Taken together, this leads to the expectation that, in societies where fathers take up a larger part of parenting, nonresident fathers are more involved with their children. Evidence also indicates that societal expectations on fathers’ participation in childrearing towards nonresident fathers are higher in countries with more paternal involvement [70, 71]. Yet, other studies suggest that expectations towards nonresident fathers are lower than those towards resident ones [72]. To shed light on the relationship between societal father practices and nonresident father-child contact, we assess whether in societies where resident fathers are more involved in childcare activities, nonresident fathers also exhibit higher levels of contact with their children than in societies where resident fathers are less involved in childcare activities. Considering fathering practices in addition to their attitudes is important as previous research has shown that attitudes do not always match actual behaviors [73, 74].

### 2.4. Child age and nonresident father-child contact

Previous studies have considered child age as an in important predictor of nonresident father-child contact [8]; however, they yield mixed findings. Some studies find that nonresident father-child contact tends to decrease with child age [3, 41, 72], while others suggest that fathers have more contact with older children as they are more at ease in interacting with older than with younger children [75]. To shed more light on whether the frequency of contact differs between older and younger children and to assess the role of individual and societal gender role attitudes as well as societal father practices for different age ranges, below we distinguish children (age 0–13) and adolescents (age 14–18).

## 3. Data and methodology

### 3.1. Data and samples

We use data from the first wave of the Generations and Gender Survey (GGS) [33, 34]. The GGS is a set of comparative surveys of nationally representative samples of the 18-79-year-old resident population, collected between 2004 and 2011. Our analysis includes 11 of the 20 countries: Austria, Belgium, Bulgaria, Czech Republic, Estonia, France, Georgia, Lithuania, Poland, Romania and Russia. The Austrian sample only includes individuals aged 18 to 45. The countries were selected based on their close-to-complete and harmonized information on nonresident father-child contact and the other variables included in the analysis. Due to missing information on key variables, we were not able to include Australia, Belarus, Germany, Hungary, Italy, Japan, the Netherlands, Norway and Sweden.

We select male respondents with at least one underage nonresident child. If a respondent reports several nonresident children, we focus on the youngest. We analyze two samples: A first sample comprises fathers with nonresident children aged 0 to 13. Fathers were asked *How often do you look after [name]*?. We call this the *childhood-sample*. Second, a subsample of fathers with children aged 14 to 17 is analyzed with respect to the question *How often do you see [name]*?. We refer to this as the *adolescence-sample*. The childhood-sample does not include Estonia and Poland; the adolescence-sample does not include Austria and Romania. To check the robustness of the results, we also run the models with only including the countries that are part of both samples.

To exclude that fathers refer to children in shared custody as nonresident children, we have verified whether fathers report resident children having the same age as the nonresident child. This applies to five cases (two aged <14; three aged 14+), which have been removed. To deal with missing values, we use multiple imputation by chained equations (MICE, Stata) with 30 imputed datasets. The childhood-sample includes 943 observations, with 809 complete cases (i.e. cases with information on all variables considered in the analysis). The following variables are imputed: Information on monthly contact (13% missing values), individual gender attitudes, gender of the nonresident child, fathers’ level of education, time since parental separation, fathers’ partnership status (all <1% missing values). The adolescence-sample consists of 549 fathers, with 460 complete cases. For the adolescence-sample, the following variables are imputed: Information on monthly contact (10% missing values), economic hardship (14%), individual gender attitudes (5%), fathers’ educational attainment (2%), time since separation and fathers’ current partnership status (<1%). As a sensitivity check we also run the models without imputed values for the outcome variables [for a recent discussion thereon see 76] and for the non-imputed data (i.e. casewise deletion).

Previous research indicates that men tend to underreport their nonresident children [77] and to overreport their involvement [36]. This implies that we may overestimate their actual involvement.

### 3.2. Measures

#### 3.2.1. Nonresident father-child contact

Fathers were asked to indicate the frequency of contact according to different time units (i.e. week, month, and year). Similarly to other studies [78–82], we consider monthly contact-frequency and use a binary indicator to distinguish fathers who do have no or very rare contact, i.e. less than once per month (0 = *no monthly contact*) and those who have contact at least once per month (1 = *monthly contact*). We have also tested models in which we distinguish three contact-categories, i.e. *never* (< once a month), *sometimes* (1–4 times per month) and *often* (more than 4 times per month) having contact. However, that indicator did not yield significant additional insights, so that we here present the results from the more parsimonious models. Information on the actual amount of time fathers spend with their children is not available in the data.

An indicator for *gender role attitudes* was constructed at the individual- and the country-level. These indicators are based on six items that have been used by previous analyses of the GGS-data [e.g. 18]. Respondents were asked to what extent they agree with the following statements (five-point scale; 1 = strongly agree; 5 = strongly disagree): (a) “In a couple it is better for the man to be older than the woman”; (b) “If a woman earns more than her partner, it is not good for the relationship”; (c) “On the whole, men make better political leaders than women”; (d) “A pre-school child is likely to suffer if his/her mother works”; (e) “If parents divorce it is better for the child to stay with the mother than with the father”; and (f) “When jobs are scarce, men should have more right to a job than women” (Cronbach’s α = .72). Based on all observations from the eleven countries (i.e. before identifying the nonresident father samples) the six items were averaged into a scale with higher scores indicating more modern gender role attitudes and lower scores indicating more traditional gender role attitudes. The societal-level indicator aims at measuring the general societal context; therefore, it has been constructed by aggregating all responses to the country-level. These indicators are standardized. To obtain measures for individual gender role attitudes, we used the responses on gender role attitudes obtained from fathers in the childhood- and adolescence-sample and standardized them for both samples. We accept up to two missing items. This choice allowed us to include Estonia for which data on item (c) and (e) were not collected.

#### 3.2.2. Societal father practices

Based on all observations from the eleven countries we have constructed an index of six items asking respondents with cohabiting children younger than 14 who carries out a specific childcare task. These tasks are “Dressing the children or seeing that the children are properly dressed”; “Putting the children to bed and/or seeing that they go to bed”; “Staying at home with the children when they are ill”; “Playing with the children and/or taking part in leisure activities with them”; “Helping the children with homework”; and “Taking the children to/from school, day care centre, babysitter or leisure activities” (Cronbach’s α = .79). These activities include physical and interactive care, which have also been considered by previous research [17, 61, 83]. Based on the responses we have constructed a two-category indicator for each task: It is mostly the mother who carries it out (= 0) or it is mostly the father who carries it out or both parents carry it out equally frequently (= 1). We have combined whether it was mostly the father or both parents equally, as the proportion of fathers who carry out the larger share is very low. Cases in which someone else or the child performs the task were not considered in the construction of this indicator. Based on the binary variable we have created an aggregate measure for each country that expresses the proportion of fathers carrying out the tasks more often or both partners’ being equally involved. This proportion ranges from .21 in Georgia to .48 in France.

#### 3.2.3. Control variables

The choice of control variables follows previous research on nonresident father-child contact. We include the *fathers’ highest educational attainment* in three categories (low (International Standard Classification of Education (ISCED) 0–2), medium (ISCED 3–4), high (ISCED 5–6)), *employment status* (employed, including self-employed vs. unemployed or not working due to any reason), whether they report experiencing *economic hardship* (= 1) or not (= 0) and their *age* in three categories (<35, 35–45, >45). These factors have been identified by prior research as important predictors of nonresident father-child contact [4, 9, 10, 28, 41, 75, 81, 84]. We also include *fathers’ present partnership status* indicating whether he is (1) not currently in a relationship, (2) in a non-cohabiting relationship or (3) in a cohabiting relationship. Furthermore, we control for having *resident children* (= 1) or not (= 0). This is important as due to competing roles and time constrains [10] separated fathers may ‘swap families’ [80, 85] and shift their investments towards their new union [4, 9, 79, 86]; particularly if new biological children arise from that union [9, 85, 87]. We also include a measure indicating the *number of years since the separation* from the child’s mother as a proxy for the time the father no longer cohabits with the child, which has been shown to relate to a decrease in contact [3, 8, 9, 36]. We control for *child age* and *gender* (0 = boy; 1 = girl).

### 3.3. Analytic strategy

First, descriptive statistics on father-child contact across countries and the variables of interest are presented. Second, logistic regression is used to assess the determinants of nonresident father-child contact. We estimate cluster-adjusted robust standard errors to take into account that individuals from the same country cannot be treated as independent observations [88–90]. We estimate four sets of models: Model 1 includes fathers’ individual-level gender role attitudes. Model 2 includes country-level gender role attitudes. In Model 3, we test how father practices relate to nonresident father-child contact. For fathers’ actual behaviors, individual gender role attitudes as well as societal expectations and norms matter [30, 54, 57, 69, 91]. Therefore, in Model 4, we test how individual gender role attitudes and (a) societal gender attitudes and (b) societal father practices affect contact. Due to their high collinearity, the two macro-level variables are not included in one model. Across models, we control for the socio-demographic variables described above and all models are estimated for the childhood- and the adolescence-sample. *No monthly contact* is the reference category, to which the category *monthly contact* is compared. The results of the logistic regression models are presented in terms of average marginal effects (AME), so that the coefficients are comparable across samples and models [92].

## 4. Descriptive results

### 4.1. Nonresident father-child contact across countries

Table 1 shows the distribution of the binary (*no monthly* vs. *monthly contact*) and the continuous monthly indicator of father-child contact for the childhood- and adolescence-sample by country. With respect to the childhood-sample, more than half of the fathers from Romania, Lithuania, Georgia and Russia do not have monthly contact. For the adolescence-sample, this only applies to fathers from Russia. Moreover, in the childhood-sample, the Western European countries rank higher in the monthly contact-category than the Eastern European countries; the only exception is Czech Republic, which is the only Eastern European country that is comparable to the Western ones in this regard. In the adolescence-sample, an Eastern-Western division is not manifested: In Austria and Belgium as well as in Georgia and Czech Republic more than 75% of the nonresident fathers have monthly contact with their adolescent children, while in the other Eastern European countries and in France, the fraction of fathers who have monthly contact is lower. Across countries and in both samples, there are on average five monthly father-child contacts (with a standard deviation of almost 8). This does not suggest that there is less contact with older children, which has been indicated by prior studies [3, 41, 72].

**Table 1:** Descriptive statistics of frequency of monthly nonresident father-child contact by country, binary and continuous measure, percentage or mean/standard deviation (complete cases).

	Childhood-sample					Adolescence-sample				
	No monthly contact (%)	Monthly contact (%)	*Mean*	*SD*	*N*	No monthly contact (%)	Monthly contact (%)	*Mean*	*SD*	*N*
Austria	17.7	82.4	5.82	7.88	102					
Belgium	16.0	84.0	7.29	6.21	75	19.4	80.6	5.58	5.71	36
Bulgaria	44.1	55.9	4.21	7.23	59	20.0	80.0	7.94	10.26	40
Czech Republic	18.9	81.1	4.75	7.09	74	25.0	75.0	4.90	7.02	40
Estonia						36.2	63.8	4.02	7.63	47
France	15.2	84.8	8.25	9.95	151	36.3	63.8	5.39	7.87	80
Georgia	59.5	40.5	4.24	6.91	42	20.0	80.0	7.35	9.21	25
Lithuania	61.3	38.7	1.78	3.86	75	42.2	57.8	3.05	5.91	45
Poland						33.3	66.7	4.96	7.89	63
Romania	73.9	26.1	1.22	3.39	46					
Russia	55.7	44.3	3.93	7.32	185	51.2	48.8	4.02	7.81	84
*Total*	*37*.*2*	*62*.*8*	*5*.*04*	*7*.*70*	809	*34*.*6*	*65*.*4*	*5*.*01*	*7*.*82*	*460*

*Source*: Gender and Generations Survey Wave 1; data before multiple imputation.

### 4.2. Gender role attitudes and father practices across countries

**Fig 1:**
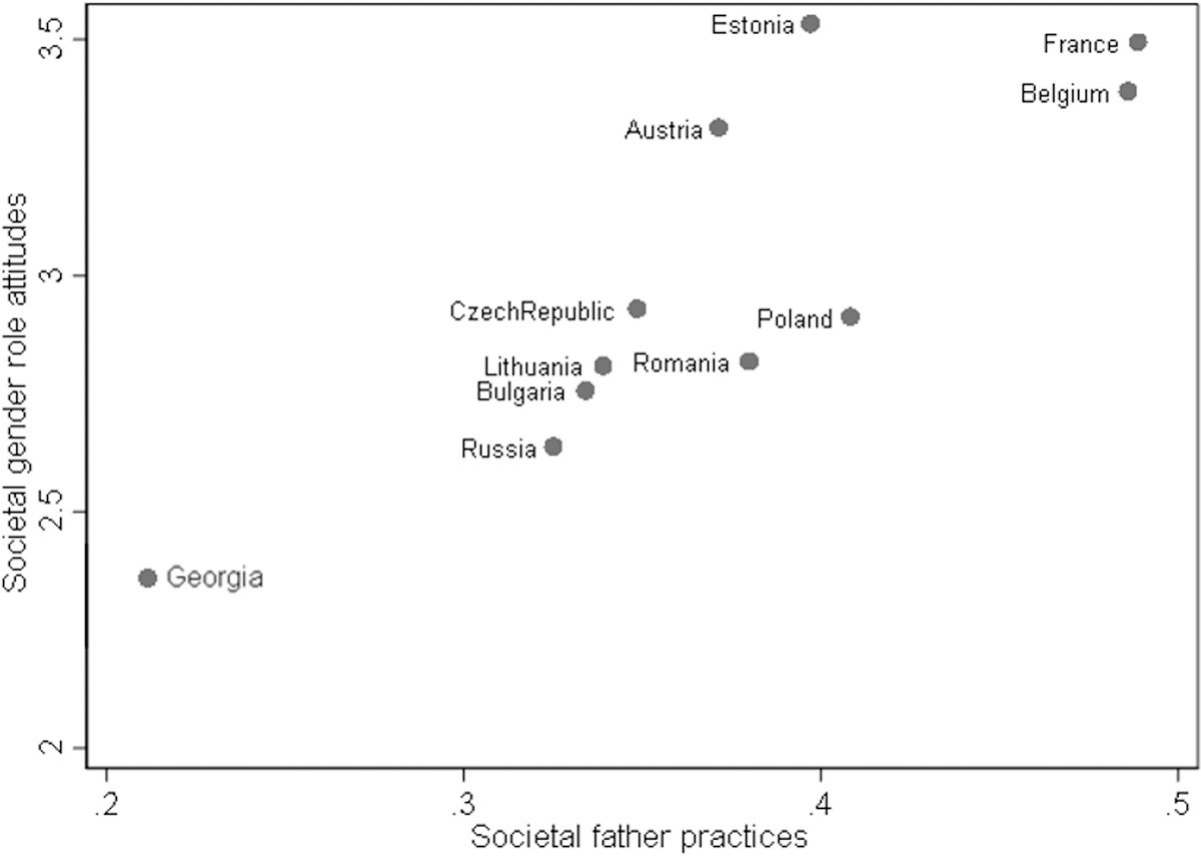
Distribution of societal gender ideologies and societal father practices, by country.

Fig 1 shows that there is variation in terms of gender ideologies as well as societal father practices across countries. Austria, France and Belgium stand out as they score high on both measures. Estonia ranks highest on egalitarian gender role attitudes and relatively high on societal father practices. Georgia ranks lowest on both measures. Table 2 displays the descriptive statistics for individual and societal gender role attitudes and societal father practices by country; the societal measures refer to both samples. Gender role attitudes are most egalitarian in Estonia, France, Belgium and Austria and lowest in Georgia and Russia. Overall, nonresident fathers’ gender role attitudes are relatively comparable to those measured at the county-level. Fathers are most involved in childcare tasks in Belgium, France and Austria.

**Table 2:** Descriptive statistics of individual gender role attitudes for the childhood- and the adolescence-sample (means and standard deviations; complete cases) and aggregated societal gender role attitudes and father practices (means).

	Individual gender role attitudes Childhood-sample			Individual gender role attitudes Adolescence-sample			Societal gender role attitudes (aggregated)	Societal father practices (aggregated)
	Mean	SD	N	Mean	SD	N	Mean	Mean
Austria	3.18	.60	102				3.31	.37
Belgium	3.48	.55	75	3.55	.62	36	3.39	.49
Bulgaria	2.70	.53	59	2.73	.46	40	2.76	.33
Czech Republic	2.91	.60	74	3.08	.58	40	2.93	.35
Estonia				3.22	.72	47	3.53	.40
France	3.62	.87	151	3.36	.88	80	3.49	.49
Georgia	2.35	.56	42	2.19	.58	25	2.36	.21
Lithuania	2.74	.57	75	2.72	.56	45	2.81	.33
Poland				2.97	.49	63	2.91	.41
Romania	2.75	.47	46				2.81	.37
Russia	2.63	.56	185	2.64	.49	84	2.64	.33
*Individual gender role attitudes*, *by sample*	*3*.*00*	.*75*	*809*	*3*.*00*	.*71*	*460*		
*Societal gender role attitudes*, *by sample*	*3*.*00*	.*37*	*809*	*3*.*01*	.*37*	*460*		
*Societal father practices*, *by sample*	.*38*	.*08*	*809*	.*38*	.*07*	*460*		

*Note*: Individual and societal gender role attitudes, min = 1, max = 5; societal father practices, min = 0, max = 1.

*Source*: Gender and Generations Survey Wave 1; data before multiple imputation.

### 4.3. Socio-demographic variables

Table 3 and Table 4 present the descriptive statistics for the childhood- and the adolescence-sample, for the full samples as well as by contact category. In both samples, we observe that fathers with more modern individual-level gender role attitudes tend to more often have monthly contact. For the childhood-sample, similar patterns are observed for societal gender role attitudes and societal father practices. Considering the father characteristics of the childhood-sample, differences between fathers not having monthly contact and those having monthly contact reveal: Being unemployed, experiencing economic hardship, having separated from the mother a longer time ago, being in a new relationship and having resident children relates to not having monthly contact. Older child-age also relates to not having monthly contact. Turning to the adolescence-sample, few characteristics differentiate fathers in the no-monthly-contact-category from those in the monthly-contact-category: Fathers experiencing economic hardship and those having separated from the child’s mother a longer time ago more often fall into the no-monthly-contact-category.

**Table 3:** Descriptive statistics for the childhood-sample (percentage or mean and standard deviation (SD)).

			Full sample	By contact category		
				No monthly contact	Monthly contact	Difference between categories
*Father characteristics*						
	Gender role attitudes		3.00 (.75)	2.77 (.69)	3.13 (.75)	***
	Educational attainment					
		Low	17.3	20.6	15.4	
		Medium	58.7	55.8	60.4	
		High	24.0	23.6	24.2	
	Employment status					
		(Self-)employed	78.0	71.4	81.9	***
		Unemployed/other	22.0	28.6	18.1	
	Economic hardship					
		Yes	64.4	76.33	57.3	***
		No	35.6	23.7	42.7	
	Age					**
		<35	45.1	50.2	42.1	
		35–45	44.1	36.5	48.6	
		>45	10.8	13.3	9.3	
	Years since separation		5.40 (3.51)	6.08 (3.71)	5.00 (3.32)	***
	Partnership status					***
		Cohabiting partner	37.0	45.9	31.7	
		Non-cohabiting partner	22.4	17.9	25.0	
		No partner	40.7	36.21	43.3	
	Co-resident children					
		Yes	26.3	33.9	21.9	***
		No	73.7	66.1	78.2	
*Child characteristics*						
	Sex					
		Boy	49.1	50.2	48.4	
		Girl	50.9	49.8	51.6	
	Age		8.41 (3.59)	9.15 (3.48)	7.98 (3.60)	***
*Country-level variables*						
	Gender role attitudes		3.00 (.37)	2.82 (.30)	3.11 (.36)	***
	Societal father practices		.38 (.08)	.35 (.06)	.40 (.08)	***
N_observations_			809	301	508	
%_observations/complete cases_			100	37.2	62.8	
N_countries_			9			

*Source*: Gender and Generations Survey Wave 1; data before multiple imputation.

*t*-tests/*chi*^*2*^-tests

*** p<0.001

** p<0.01

* p<0.05

**Table 4:** Descriptive statistics for the adolescence-sample (percentage or mean and standard deviation (SD)).

			Full sample		By contact category		
				Difference between full samples	No monthly contact	Monthly contact	Difference between categories
*Father characteristics*							
	Gender role attitudes		2.97 (.71)		2.85 (.70)	3.03 (.71)	*
	Educational attainment						
		Low	16.1		16.4	16.0	
		Medium	61.5		61.0	61.8	
		High	22.4		22.6	22.3	
	Employment status						
		(Self-)employed	70.7	**	67.9	72.1	
		Unemployed/other	29.4		32.1	27.9	
	Economic hardship						
		Yes	69.5		76.8	65.7	*
		No	30.5		23.2	34.3	
	Age			***			
		<35	4.4		6.3	3.3	
		35–45	54.6		55.4	54.2	
		>45	41.1		38.4	42.5	
	Years since separation		10.33 (5.13)	***	11.33 (4.59)	9.80 (5.32)	**
	Partnership status			***			
		Cohabiting partner	50.7		47.8	52.2	
		Non-cohabiting partner	12.8		13.8	12.3	
		No partner	36.5		38.4	35.6	
	Co-resident children						
		Yes	31.3		32.1	30.9	
		No	68.7		67.9	69.1	
*Child characteristics*							
	Sex						
		Boy	52.4		56.0	50.5	
		Girl	47.6		44.0	49.5	
	Age		15.97 (1.16)	***	15.93 (1.21)	16.00 (1.13)	
*Country-level variables*							
	Gender role attitudes		3.01 (.37)		3.00 (.37)	3.02 (.37)	
	Societal father practices		.38 (.07)		.38 (.07)	.38 (.08)	
N_obs_			460		159	301	
%_obs/complete cases_			100		34.6	65.4	
N_countries_			9				

*Source*: Gender and Generations Survey Wave 1; data before multiple imputation.

*t*-tests/*chi*^*2*^-tests

*** p<0.001

** p<0.01

* p<0.05

Tables 3 and 4 also bring out the comparability of the childhood- and the adolescence-sample. Despite their general comparability, some differences reveal: Compared to fathers in the adolescence-sample, a higher proportion of fathers in the childhood-sample is employed. Fathers in the childhood-sample are younger and have separated from the mother more recently. Fathers in the childhood-sample less often have a cohabiting partner.

## 5. Multivariate results

We now present the results from the logistic regressions. The results for the childhood-sample are presented in Table 5 and those for the adolescence-sample in Table 6. In all models, the reference category is *no monthly contact* to which *monthly contact* is compared; i.e., the AMEs show the probability of monthly contact.

**Table 5:** Average marginal effects from the logistic regression predicting monthly nonresident father-child contact for the childhood-sample (reference: no monthly contact).

				Model 1	Model 2	Model 3	Model 4a	Model 4b
*Variables of interest*								
	Individual gender role attitudes			0.445***			0.115	0.194
				(0.093)			(0.092)	(0.105)
	Societal gender role attitudes				0.852***		0.800***	
					(0.087)		(0.097)	
	Societal father practices					0.713***		0.626***
						(0.111)		(0.121)
*Father characteristics*				ref.	ref.	ref.	ref.	ref.
	Educational attainment	Low		-0.381	-0.644**	-0.579*	-0.652**	-0.591*
				(0.240)	(0.243)	(0.247)	(0.239)	(0.241)
		Medium		ref.	ref.	ref.	ref.	ref.
		High		-0.068	0.044	-0.014	0.037	-0.018
				(0.276)	(0.240)	(0.252)	(0.241)	(0.255)
	Employment status							
		(Self-)employed						
		Unemployed/other		-0.423*	-0.319	-0.333*	-0.310	-0.322*
				(0.165)	(0.171)	(0.158)	(0.171)	(0.160)
	Economic hardship			-0.589*	-0.400	-0.571**	-0.387	-0.533*
				(0.263)	(0.205)	(0.212)	(0.209)	(0.219)
	Age							
		<35		-0.574*	-0.366	-0.447	-0.372	-0.450
				(0.273)	(0.246)	(0.259)	(0.248)	(0.258)
		35–45		ref.	ref.	ref.	ref.	ref.
		>45		-0.525	-0.779	-0.848	-0.779	-0.832
				(0.398)	(0.450)	(0.478)	(0.449)	(0.473)
	Years since separation			-0.036	-0.050	-0.040	-0.052	-0.042
				(0.027)	(0.033)	(0.029)	(0.033)	(0.029)
	Partnership status							
		Cohabiting partner		ref.	ref.	ref.	ref.	ref.
		Non-cohabiting partner		0.464	0.654*	0.540	0.653*	0.546
				(0.281)	(0.282)	(0.283)	(0.287)	(0.291)
		No partner		0.479*	0.566**	0.523*	0.563**	0.520*
				(0.190)	(0.205)	(0.209)	(0.204)	(0.208)
	Any co-resident children			-0.168	-0.033	-0.093	-0.040	-0.101
				(0.216)	(0.275)	(0.256)	(0.272)	(0.255)
*Child characteristics*								
	Boy			-0.075	-0.118	-0.098	-0.109	-0.085
				(0.165)	(0.143)	(0.140)	(0.145)	(0.145)
	Age			-0.088***	-0.054*	-0.073**	-0.054*	-0.072**
				(0.021)	(0.023)	(0.024)	(0.024)	(0.024)
N_observations_				943				
N_countries_				9				

*Source*: GGS, Wave 1

Robust standard errors in parentheses.

Multiply imputed data, Rubin’s rules apply.

*** p<0.001

** p<0.01

* p<0.05

**Table 6:** Average marginal effects from the logistic regression predicting monthly nonresident father-child contact for the adolescent-sample (reference: no monthly contact).

				Model 1	Model 2	Model 3	Model 4a	Model 4b
*Variables of interest*								
	Individual gender role attitudes			0.249			0.281*	0.325*
				(0.153)			(0.128)	(0.143)
	Societal gender role attitudes				0.058		-0.072	
					(0.170)		(0.156)	
	Societal father practices					-0.021		-0.175
						(0.153)		(0.140)
*Father characteristics*								
	Educational attainment							
		Low		0.042	0.035	0.069	0.070	0.116
				(0.250)	(0.245)	(0.245)	(0.243)	(0.248)
		Medium		ref.	ref.	ref.	ref.	ref.
		High		-0.234	-0.205	-0.216	-0.248	-0.256
				(0.180)	(0.199)	(0.199)	(0.188)	(0.187)
	Employment status							
		(Self-)employed		ref.	ref.	ref.	ref.	ref.
		Unemployed/other		-0.143	-0.169	-0.168	-0.139	-0.133
				(0.209)	(0.207)	(0.206)	(0.208)	(0.205)
	Economic hardship			-0.421*	-0.471*	-0.519*	-0.454*	-0.507*
				(0.210)	(0.228)	(0.226)	(0.217)	(0.219)
	Age			ref.	ref.	ref.	ref.	ref.
		<35		-0.510	-0.500	-0.516	-0.524	-0.553
				(0.382)	(0.383)	(0.385)	(0.385)	(0.395)
		35–45		ref.	ref.	ref.	ref.	ref.
		>45		0.164	0.102	0.122	0.188	0.235
				(0.163)	(0.179)	(0.179)	(0.169)	(0.168)
	Years since separation			-0.074**	-0.075***	-0.076***	-0.074**	-0.073**
				(0.023)	(0.022)	(0.021)	(0.023)	(0.023)
	Partnership status							
		Cohabiting partner		ref.	ref.	ref.	ref.	ref.
		Non-cohabiting partner		-0.344	-0.358	-0.352	-0.341	-0.296
				(0.223)	(0.224)	(0.221)	(0.222)	(0.217)
		No partner		-0.345	-0.361	-0.343	-0.330	-0.298
				(0.206)	(0.212)	(0.212)	(0.205)	(0.207)
	Any co-resident children			-0.094	-0.129	-0.122	-0.082	-0.070
				(0.211)	(0.205)	(0.201)	(0.221)	(0.219)
*Child characteristics*								
	Boy			-0.268	-0.268	-0.263	-0.263	-0.258
				(0.256)	(0.265)	(0.266)	(0.260)	(0.258)
	Age			0.089	0.101	0.102	0.087	0.086
				(0.092)	(0.088)	(0.088)	(0.093)	(0.093)
N_observations_				549				
N_countries_				9				

*Source*: GGS, Wave 1

Robust standard errors in parentheses.

Multiply imputed data, Rubin’s rules apply.

*** p<0.001

** p<0.01

* p<0.05

## 5.1. Gender role attitudes and societal father practices

First, we focus on how individual gender role attitudes relate to nonresident father child-contact (Model 1). Concerning the childhood-sample, we find that more modern gender role attitudes relate to an increased probability of having monthly contact compared to rather traditional gender role attitudes. This is different for the adolescence-sample: Individual gender role attitudes do not predict monthly nonresident father-child contact. Moreover, compared to the childhood-sample, the AME is considerably smaller.

Model 2 investigates the relationship between societal-level gender role attitudes and nonresident father-child contact. For the childhood-sample, more modern societal gender role attitudes predict the probability of monthly contact, while this is not the case with respect to fathers’ involvement with adolescent children. This suggests that more gender-egalitarian contexts favor nonresident father-child contact with younger children but not with adolescent children.

Next, we assess whether higher levels of societal father practices relate to monthly nonresident father-child contact (Model 3). For the childhood-sample, more active societal father practices predict a higher probability of monthly father-child contact. Again, this is not the case for the adolescence-sample. Taken together, Model 1, 2 and 3 suggest that individual as well as societal gender role attitudes and father practices predict a higher probability of monthly contact with children but not with adolescents. However, Models 1, 2 and 3 do not consider individual- and societal-level predictors jointly; in the following sections, we will analyze them simultaneously.

### 5.2. Individual gender role attitudes and societal indicators considered jointly

The literature [57, 69, 91], as well as the above results for the childhood-sample suggest that both individual gender role attitudes as well as societal gender role attitudes and societal father practices affect the probability of contact. Therefore, in what follows, we consider models accounting for individual gender role attitudes and the societal-level indicators. Given a relatively high collinearity between both societal indicators, we do not introduce them in the same model.

Model 4a assesses how individual and societal gender role attitudes jointly affect nonresident father-child contact, i.e. we include them in the model simultaneously. First, we focus on the childhood-sample. Individual gender role attitudes no longer predict nonresident father-child contact, when also accounting for societal gender role attitudes. Compared to Model 1, in Model 4a, the coefficient for individual gender role attitudes becomes insignificant and the AME is considerably smaller. More egalitarian societal gender role attitudes predict a higher probability of monthly contact and the AME is similar to that in Model 2. This indicates that for the childhood-sample individual gender role attitudes are less decisive for monthly contact than societal gender role attitudes. Regarding the adolescence-sample, a different pattern reveals. The AME for societal gender role attitudes is negligible, while that for individual gender role attitudes slightly increases compared to Model 1 and becomes statistically significant. Hence, more egalitarian individual gender role attitudes predict an increased probability of monthly contact. This suggests that societal gender role attitudes do not influence the probability of monthly contact between nonresident fathers and their adolescent children, while individual-level gender role attitudes predict contact.

Model 4b, analyses individual gender role attitudes jointly with societal father practices. For the childhood-sample, the results from Model 3 are confirmed: More intense societal father practices predict a substantially higher probability of monthly contact. Compared to Model 1, individual gender role attitudes are no longer indicative of a higher monthly contact probability. Hence, involving fathers more into general childrearing seems beneficial for father-child involvement beyond union dissolution. Turning to the adolescence-sample, Model 4b shows that more egalitarian individual-level gender role attitudes increase the probability of monthly contact, while societal father practices do not predict monthly contact.

Overall, these results indicate that the societal context is more important for father-child contact with younger children, while individual gender role attitudes matter more for contact with adolescents. Moreover, Model 4a and 4b also show that when analyzing nonresident father-child contact, it is important to take into account both individual-level gender role attitudes and societal factors.

For both samples, we have also tested Models 4a and 4b with interactions between individual gender role attitudes and the societal indicators. The results reveal that there is no interaction effect between those indicators.

### 5.3. Socio-demographic characteristics

The results also yield valuable insights concerning fathers’ and children’s socio-demographic characteristics. With respect to the childhood-sample, we find that fathers who have a low educational attainment, are unemployed, experience economic hardship and, to some extent those who are younger than 35, tend to have a lower probability of monthly contact. This coincides with previous research [e.g. 4, 9, 10, 11, 23]. Not having a partner is related to a higher occurrence of monthly contact; that tendency also reveals with respect to having a non-cohabiting partner. This confirms previous studies showing that a new partnership relates to less contact between nonresident fathers and their children [e.g. 79]. The probability of monthly contact also decreases with increasing child age, an observation that has been highlighted by prior studies [3, 41, 72]. For the adolescence-sample, economic hardship and time since the parental separation predict a lower probability of monthly contact. The latter confirms previous studies, showing that nonresident fathers’ level of contact decreases with more time elapsed since parental partnership dissolution [75, 80, 93].

For neither sample, having resident children significantly relates to the frequency of contact. This is in line with some research [6], while other studies find a relationship [9, 85, 87]. The child’s gender does not predict the probability of monthly contact; again, this confirms some studies [75, 94], while others find that nonresident fathers have more contact with boys than with girls [e.g. 87, 95].

### 5.4. Robustness checks

In order to verify the robustness of the above results, we have carried out several sensitivity analyses. The most important ones are reported here. Compared to the main results, there are differences in terms of sample size; therefore, it is important to consider the AMEs. Unless stated differently, in the robustness analyses, we use multiple imputation.

The first robustness analysis considers children of different ages. Above, we have distinguished the age groups childhood and adolescence; however, the childhood-sample still includes a relatively wide age-span. As prior studies have shown that nonresident father child-contact changes with child age [3, 41, 72] and, as the main results reveal different patterns for the childhood- and adolescence-sample, we now assess if the above results hold across different ages of childhood. Therefore, we divide the childhood-sample into a subsample of children aged 0–5 (pre-school age, n = 251) and 6–13 (school age, n = 692). With respect to Model 1, individual gender role attitudes significantly predict monthly contact for school age children, but not for pre-school children (due to the smaller sample size); for school age children, the AME is somewhat bigger and closer to the main results compared to pre-school children. For both age groups, the results from Models 2, 3, 4a, 4b correspond to those from the main models, with somewhat larger AMEs for the school age as compared to pre-school age children. Societal gender role attitudes and father practices predict monthly nonresident father-child contact across childhood, while individual gender role attitudes hardly play a role.

As a second sensitivity analysis, we analyze only those countries that are part of the childhood- and the adolescence-sample (i.e. Belgium, Bulgaria, Czech Republic, Georgia, France, Lithuania, Russia; n = 792 for the childhood-sample; n = 411 for the adolescence-sample). For the childhood-sample, the results are in line with the main findings. For the adolescence-sample, the probability of monthly contact is not significantly predicted by any of the variables of interest. This is different from the findings in Model 4a and 4b, in which individual gender role attitudes significantly predict monthly contact. Yet, the AMEs for individual gender role ideologies in the main models and those in this robustness check are almost identical. Hence, the results are not driven by a specific combination of countries included in the analyses samples.

In the next set of robustness analyses, we use casewise deletion instead of multiple imputation (n = 809 for the childhood-sample; n = 460 for the adolescence-sample). The results for the childhood-sample differ in Model 4b in the sense that individual gender role ideologies now are statistically significant; yet, the AME is almost identical to the main model, confirming the main result. The results for the adolescence-sample correspond to the main analyses.

Finally, we have run the models without imputing the outcome variables [76], i.e. cases with missing data on the dependent variable were used in the imputation equation, but not in the analysis (n = 819 for the childhood-sample; n = 467 for the adolescence-sample). The results for the childhood-sample correspond to the main results, the only exception is that individual-level gender role attitudes significantly predict monthly contact in Model 4b; however, again the AME is comparable to the main model. For the adolescence-sample, the results are consistent with the main results.

Overall, the sensitivity analyses indicate that the results are robust over several model specifications; differences in terms of statistical significance do not matter much substantially.

## 6. Conclusion and discussion

Based on the GGS-data this study has analyzed how individual and societal gender role attitudes as well as societal father practices relate to the probability of monthly nonresident father-child contact. We have distinguished fathers of children aged 0 to 13 and those of children aged 14 to 17. The analyses include eleven Western and Eastern European countries.

Previous literature has established the socio-demographic characteristics that are crucial predictors of the frequency of nonresident father-child contact [9–11, 23, 79]. Our study provides evidence that in addition to those factors, individual and societal gender role attitudes and father practices relate to nonresident father-child contact and should be included in analyses thereof. Moreover, we show that contact is affected by somewhat different factors depending on whether contact between nonresident fathers and children or adolescents is considered.

More egalitarian societal gender role attitudes and more intense societal father practices predict an increased probability of nonresident father-child contact with children aged 0 to 13, while their individual gender role attitudes do not matter much. This in line with Gaunt’s [67] study on resident fathers indicating that their gender ideologies do not predict their childcare involvement; she found that instead parents’ attitudes towards fathers’ roles matter. The latter should be investigated by future studies on nonresident father-child relationships. The positive association between societal gender role attitudes and nonresident father-child contact supports Kalmijn’s [57] assumption that in countries with more traditional gender roles, fathers have less contact with their children than in countries with more egalitarian gender roles. Our finding that in countries where resident fathers are more involved in childcare, nonresident fathers are more often involved with young children, suggests that intensive fatherhood practices positively affect fathers’ role in their children’s lives beyond parental union dissolution. This might also (partly) explain the observed difference in frequency of father-child contact between Eastern and Western European countries. In most Eastern European countries, fathers tend to be less encouraged to participate in caring activities than in Western European countries where caring activities are less gendered [71].

With respect to adolescent children, another pattern reveals: More egalitarian individual gender role attitudes predict a higher probability of monthly contact, while societal factors hardly play a role. This extends the literature on resident fathers showing that egalitarian gender ideologies affect parenting behaviors and that fathers who have more egalitarian gender ideologies are more involved in their children lives [48, 49].

Overall, societal gender role attitudes as well as father practices are related to contact with younger children, but are unrelated to contact with adolescent children. It is important to underline that the average number of contacts is similar in both samples (around five per month). Hence, we do not observe a general decline in contact frequency for older children, as one may assume based on previous studies [3, 41]. Furthermore, this is not in line with the general observation that when children get older, parents spend less time in direct interaction with their children [96, 97]. Several reasons might explain why societal gender role attitudes and fathering practices are related to higher levels of contact with younger children but not with adolescents. One potential reason is that expectations towards fathers of younger children are higher than those towards older children. Moreover, there may be a generational pattern: Fathers in the childhood-sample are considerably younger than those in the adolescence-sample, and, therefore, more likely to fall into the category and culture of ‘new fatherhood’ [14, 16, 61]. Recently, gender role attitudes and fathers’ involvement in childcare have advanced, as have public policies in those domains [30, 60, 98]. Indeed, nonresident fathers’ individual gender role attitudes are slightly lower in the adolescence- than in the childhood-sample. Another potential explanation is that for the older sample, contact-patterns between fathers and their children have already been established during childhood and are not, or no longer, influenced by attitudes and societal norms and expectations [99]. If this assumption holds, it would indicate that the early years of children’s lives are crucial for contextual factors to play a role in the father-child relationship and that policies should focus on that period of life. Unfortunately, with the GGS-data we are not able to evaluate these mechanisms. Future research should consider them and longitudinal data on fathers’ pre- and post-separation behaviors should be collected.

We complement previous research that has shown that resident father-child relationships are affected by societal gender role attitudes [30, 35, 100] and extend those results to nonresident father-child relationships: Gender role attitudes, at the individual and the societal level, relate to fathers’ involvement in their resident as well as nonresident children’s lives.

Despite its contributions, our study has some noteworthy limitations. First, fathers’ pre-separation individual-level involvement in childcare is likely to predict their post-separation involvement [7, 8, 66, 98]. We are not able to account for this, as the data do not contain retrospective information about fathers’ involvement in their children’s lives during cohabitation. In the future, it would be important to shed light on how resident fathers’ involvement affects the father-child relationship after parental union dissolution. Second, as most studies, we have focused on face-to-face contact [4, 10, 78, 79, 82, 101]. Information on other types of contact such as overnight stays [8, 11, 102] or phone contact [79, 101] is not available. Yet, face-to-face contact is a condition for other types of contact [103]; therefore, we assume that our measures capture some of the variation in other forms of contact. Nevertheless, more varied measures should be included in future data collections. Third, we have taken the father’s perspective, which is a crucial contribution to the existing literature [29, 35, 36]. However, we do not have mothers’ reports on the contact frequency, which might differ as men tend to over-report their involvement; either due to recall error or social desirability [17, 36]. Furthermore, the data does not contain information on mothers’ re-partnering status and whether the child has a stepfather, who may substitute the biological father [28]. Future data collections should involve perspectives and attitudes of both parents and ideally also from the child. The focus of this study was on the quantity of father-child-contact. Future studies should also analyze the quality of the father-child-relationship.

Finally, we conclude with some policy-related considerations. Policies aiming at increasing gender equality might favor nonresident father-child contact, in particular at younger ages. Taken together with the existing literature our findings suggest that egalitarian gender role attitudes and fathering practices do not only relate to resident fathers’ involvement but also to nonresident fathers’ involvement in their children’s lives. In countries with a stronger participation of fathers in childrearing, nonresident father-child contact with younger children is more common. Therefore, promoting equal parenting seems to be a promising way to not only increase resident fathers’ involvement in their children’s lives, but also that of nonresident fathers. Offering or extending fathers’ leave around childbirth, providing free childcare and the right to request flexible working hours may be potential ways [17, 56, 61, 104].
